# Relationship between adenovirus DNA replication proteins and nucleolar proteins B23.1 and B23.2

**DOI:** 10.1099/vir.0.83196-0

**Published:** 2007-12

**Authors:** Clemence E. Hindley, Andrew D. Davidson, David A. Matthews

**Affiliations:** Department of Cellular and Molecular Medicine, University Walk, University of Bristol, Bristol BS8 1TD, UK

## Abstract

Adenovirus infection subverts nucleolar structure and function. B23 is a nucleolar protein present in two isoforms (B23.1 and B23.2) and both isoforms have been identified as stimulatory factors for adenovirus DNA replication. Here, it is demonstrated that the two isoforms of B23, B23.1 and B23.2, interact and co-localize differently with viral DNA replication proteins pTP and DBP in adenovirus-infected cells. Thus, the mechanism by which the two proteins stimulate viral DNA replication is likely to differ. These data also demonstrate the importance of testing both isoforms of B23 for interactions with viral proteins and nucleic acids.

Nucleolar protein B23 exists as two isoforms that are present in similar amounts, differing only at their carboxy termini (Chang & Olson, 1989; Wang *et al.*, 1993). B23.1 is 294 aa long and is known to participate in the infectious cycle of diverse viruses, including adenovirus (Okuwaki *et al.*, 2001), adeno-associated virus (Bevington *et al.*, 2007), human immunodeficiency virus type 1 (Fankhauser *et al.*, 1991; Flint & Shenk, 1997), hepatitis C virus (Mai *et al.*, 2006) and Japanese encephalitis virus (Tsuda *et al.*, 2006). In contrast, B23.2 is 259 aa long, sharing the first 257 aa with B23.1, and is derived by differential splicing. Its role in the replication of any virus other than adenovirus has not been examined.

The icosahedral adenovirus particle contains a double-stranded DNA genome approximately 36 kbp in size. Replication in the cell nucleus results in nucleolar disruption (Castiglia & Flint, 1983) and redistribution of nucleolar antigens, including B23.1 (Lutz *et al.*, 1996; Matthews, 2001; Walton *et al.*, 1989) and upstream binding factor (UBF) (Lawrence *et al.*, 2006). Previous research showed that B23.1 and B23.2 stimulate adenovirus DNA replication independently (Okuwaki *et al.*, 2001) and concluded that the two proteins are essentially equivalent in their activities, perhaps acting as molecular chaperones. Three viral proteins are central to adenovirus DNA replication: the viral polymerase (Adpol), preterminal protein (pTP), which primes DNA synthesis (Liu *et al.*, 2003), and DNA-binding protein (DBP), which binds single-stranded DNA displaced during genome replication and is required for initiation and elongation of replication (de Jong *et al.*, 2003b). During infection, a virally encoded protease cleaves pTP (653 aa) to produce mature TP via an intermediate known as iTP, eventually removing 350 aa from the N terminus (Webster *et al.*, 1994).

Here, we show that B23.1 interacts with both pTP and DBP, whereas B23.2 interacts with DBP only in *in vitro* pull-down assays. Early in infection, DBP accumulates in discrete areas in the nucleus and recruits B23.2 only. Later, pTP becomes detectable in separate centres adjacent to DBP. At this stage, both B23.1 and B23.2 are recruited to the pTP-rich centres only.

A pull-down assay was used to identify adenovirus DNA replication proteins that interact with human B23.1 and B23.2. Both sequences were amplified from pGFP-B23 (Chen & Huang, 2001), inserted into pRSETA (Invitrogen) and expressed with an N-terminal His_6_ tag. Constructs were transformed into BL21-Gold (DE3) pLysS competent *Escherichia coli* and, following induction with 0.1 mM IPTG, proteins were purified by using Ni–NTA agarose (Qiagen) and eluted with 250 mM imidazole. Purified proteins were coupled onto CNBr-activated Sepharose (Sigma) following the manufacturer's instructions. Approximately 10^7^ HeLa cells were infected with human adenovirus serotype 2 (Ad2) at an m.o.i. of 5. At 18 h post-infection, duplicate cell extracts were prepared by sonication of the cells in PBS/1 % NP-40 (v/v) and passed over equivalent amounts (50 μg) of immobilized B23.1 or B23.2. Following PBS washes, bound proteins were eluted in 2× SDS-PAGE loading buffer and subjected to Western blotting with antibodies against the viral replication proteins pTP [mouse 3D11 (Webster *et al.*, 1997); Fig. 1a[Fig f1]] and DBP (a kind gift of Professor W. Russell, University of St Andrews, UK; Fig. 1b[Fig f1]). We found that B23.1 (but not B23.2) interacted with pTP; there was reduced interaction with iTP and essentially none with TP in this experiment. Relative to iTP and TP, pTP has a higher affinity for viral DNA and it has been proposed that pTP helps to stabilize the interaction between the viral polymerase and partially unwound viral DNA at the origin (de Jong *et al.*, 2003a). As pTP interacts with viral DNA at the origins of replication, this may indicate a direct stimulatory role for B23.1 at this step in viral DNA replication (de Jong *et al.*, 2003a, b; Webster *et al.*, 1994). B23.1 and B23.2 interacted with DBP, but neither isoform bound Adpol (data not shown). For a reverse pull-down, pTP and DBP were baculovirus-expressed and purified as described previously (Monaghan *et al.*, 1994; Temperley & Hay, 1992), then immobilized on Sepharose columns. The columns were used to bind proteins from uninfected cell lysates and the bound proteins were probed in a Western blot with anti-B23.1 (Zymed) or anti-B23 (Santa Cruz), which detects both isoforms. In the absence of other viral proteins and/or DNA, B23.1 associated with pTP, but not DBP (Fig. 1c[Fig f1]). We saw no association of DBP with B23.1 or B23.2 (data not shown), indicating that this interaction may be dependent upon additional viral factors. Fig. 1(d) shows a Coomassie stain of the purified B23.1 and B23.2 used in these experiments. Confirmation that the interaction between B23.1 and pTP was direct was gained by means of an immunoprecipitation assay in which anti-B23.1 and anti-pTP antibodies, in combination with protein G–agarose (Sigma), both precipitated B23.1 from a mixture containing only recombinant B23.1 and pTP (Fig. 1e[Fig f1]). However, when pTP was omitted from the mixture, the anti-pTP antibody did not precipitate B23.1.

We then compared the localization of B23.1 and B23.2 with that of pTP and DBP in infected cells. As there is no B23.2-specific antibody, the localization of B23.1 and B23.2 was examined by using constructs encoding B23.1 and B23.2 tagged N-terminally with either enhanced green fluorescent protein (EGFP) or Myc. HeLa cells (grown on glass coverslips in Dulbecco's modified Eagle's medium supplemented with 10 % fetal calf serum) were either transfected with 1 μg plasmid DNA by using Lipofectamine 2000 (Invitrogen) and infected with Ad2 at an m.o.i. of 5, or were infected only. The localization of B23.1, B23.2, pTP and DBP was then examined by immunofluorescence at 18 h post-infection.

Examining B23.2 first, we saw that EGFP–B23.2 was localized mainly in the nucleolus of uninfected cells, as indicated by the nucleolar antigen nucleolin (Fig. 2a[Fig f2]). As adenovirus infection began and significant levels of DBP accumulated, we saw movement of some EGFP–B23.2 from the nucleolus into DBP-rich centres (Fig. 2b[Fig f2]). Fig. 2(c) shows two infected cells transfected with EGFP–B23.2, the one on the left expressing pTP and DBP and the one on the right expressing DBP only. The left-hand cell represents a later stage in the infection, when there is a greater number of smaller DBP-rich centres alongside newly formed pTP-rich centres. This is typical of the timing of the detection of these antigens in an adenovirus infection when a wider range of times are examined (i.e. DBP detection prior to pTP detection; data not shown) and illustrative that these infections are asynchronous. We saw that, in cells lacking detectable pTP, EGFP–B23.2 colocalized with DBP. However, when cells expressed both DBP and pTP, EGFP–B23.2 co-localized exclusively with pTP adjacent to the DBP-rich centres.

Moving on to B23.1, a proportion of EGFP–B23.1 moved from a nucleolar location in uninfected cells (Fig. 2d[Fig f2]) to distinct nucleoplasmic spots during infection, co-localizing with pTP within the nucleoplasm [Fig. 2e[Fig f2](i)]. The movement of B23.1 from the nucleolus into the nucleoplasm only occurred once pTP was expressed and was not seen in the presence of DBP only (compare left-hand cell with right-hand cell). The same was also seen with Myc-tagged B23.1 (data not shown) and supported the pull-down data, showing that B23.1 interacts with pTP (Fig. 1a[Fig f1]). We reported previously that B23.1 has a distinct pattern in infected cells (Matthews, 2001), and Okuwaki *et al.* (2001) suggested some co-localization of haemagglutinin-tagged B23.1 with DBP in infected cells. We found that endogenous B23.1 occupied locations adjacent to DBP within the nucleoplasm, with only minor overlap (Fig. 2f[Fig f2]). The sequestration of some B23.1 into the nucleoplasm corresponded with the development of a large number of smaller DBP-rich centres [compare the two cells in Fig. 2(f)]. Indeed, in contrast to B23.2, we only observed B23.1 outside the infected-cell nucleolus once pTP expression was detectable (Fig. 2e[Fig f2]).

As endogenous levels of B23.1 and B23.2 are similar in normal cells, we also examined cells in which both Myc–B23.1 and EGFP–B23.2 were overexpressed. In infected cells, the sequestration of EGFP–B23.2 into extranucleolar sites reminiscent of DBP was unaffected by co-expression with Myc–B23.1 (Fig. 2g[Fig f2]).

We also noted that initial co-localization of EGFP–B23.2 with DBP occurred independently of endogenous B23.1, which was still nucleolar at this time (Fig. 2h[Fig f2]). However, once endogenous B23.1 was detected outside the nucleolus, EGFP–B23.2 and endogenous B23.1 both co-localized in centres distinct from DBP (Fig. 2j[Fig f2]).

Based on our finding that B23.2 interacts only with DBP from virally infected cells in pull-down assays, we propose that B23.2 is initially sequestered into DBP/viral DNA-rich centres prior to detectable pTP expression. Once pTP expression becomes detectable, B23.1 can be detected in pTP-rich centres, due to direct interaction with pTP. B23.2 is then drawn into the pTP-rich centres by interaction with the pTP–B23.1–viral DNA complex. Both isoforms have been shown, using *in vitro* assays, to stimulate viral DNA replication independently (Lawrence *et al.*, 2006; Okuwaki *et al.*, 2001); our data indicate that the two isoforms of B23 operate in two distinct replicative environments. The first is B23.2- and DBP-rich, giving way to a second environment rich in pTP, B23.1 and B23.2.

Okuwaki *et al.* (2001) presented data showing that the first 160 aa of B23 stimulated adenovirus replication *in vitro*. We investigated this mutant's ability to interact with pTP and DBP by pull-down assay, as described for B23.1. We found that B23 1–160 bound DBP and pTP (Fig. 3a, b[Fig f3]). Whilst we were unable to clone EGFP–B23 1–160, we were able to generate a Myc-tagged version that also co-localized only with pTP in infected cells (Fig. 3c[Fig f3]). A smaller mutant, 1–116 (which does not stimulate viral DNA replication), was expressed with an N-terminal EGFP tag and it too co-localized only with pTP (data not shown). This implies that the interaction of B23.1 with pTP can be separated from the stimulation of DNA replication. A Coomassie stain of the purified B23 1–160 can be seen in Fig. 3(d).

Whilst B23.2 contains the required sequences to pull down pTP from infected cells, no interaction was detected, suggesting that the conformation of B23.2 specifically prevents interaction with pTP. Our findings are summarized in Fig. 3(e).

Although B23.1 and B23.2 form multimeric complexes, we have separated the two proteins *in situ* in infected cells, reflecting the dynamic interaction between the two (Okuwaki *et al.*, 2002). However, further conclusions about B23.2 are hampered by the lack of published information on its functions in the cell and the lack of a specific antibody.

B23.1 and B23.2 affect replication of adenovirus genomes in different assays (Lawrence *et al.*, 2006; Okuwaki *et al.*, 2001). Our data show that these effects are mediated primarily through interactions with pTP and DBP, that B23.1 and B23.2 act in different replicative environments as the infection progresses and that the two isoforms have different interactions with the replicative machinery.

Three nucleolar antigens are now known to associate with the adenovirus DNA replication machinery: B23.1, B23.2 and UBF (Lawrence *et al.*, 2006). This report expands the conclusion that the nucleolus is a source of cellular co-factors for adenoviral replication and underlines the significance of B23.2 when examining the role of the nucleolus in viral replication.

## Figures and Tables

**Fig. 1. f1:**
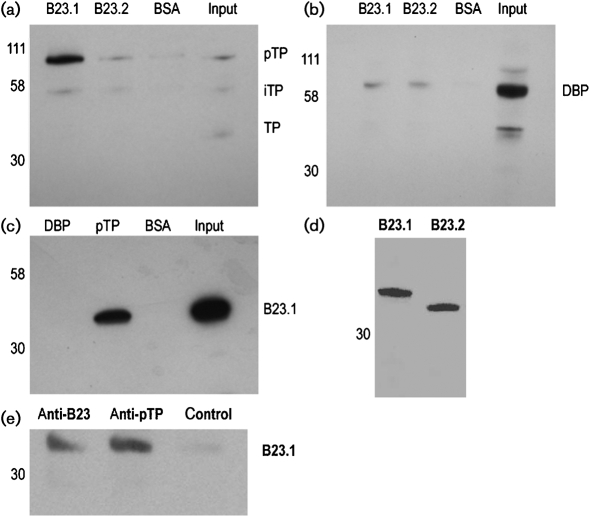
(a, b) Ad2-infected HeLa cell lysates were passed over B23.1-, B23.2- or BSA-conjugated Sepharose beads. Proteins bound to the beads were subjected to SDS-PAGE and probed by Western blot for pTP (a) and DBP (b). (c) DBP-, pTP- and BSA-conjugated beads were also used to bind proteins from uninfected HeLa cells and probed for B23.1. In (a–c), BSA-conjugated Sepharose acted as a negative control for non-specific binding. (d) Purified, His-tagged B23.1 and B23.2 were analysed by SDS-PAGE and Coomassie stain for total protein content. (e) A mixture of B23.1 and pTP were immunoprecipitated by using antibodies to either B23.1 or pTP and the proteins pulled down were probed by Western blot for B23.1. In the control, a sample of B23.1 only was immunoprecipitated by using antibody to pTP. Sizes of molecular mass markers (in kDa) are shown.

**Fig. 2. f2:**
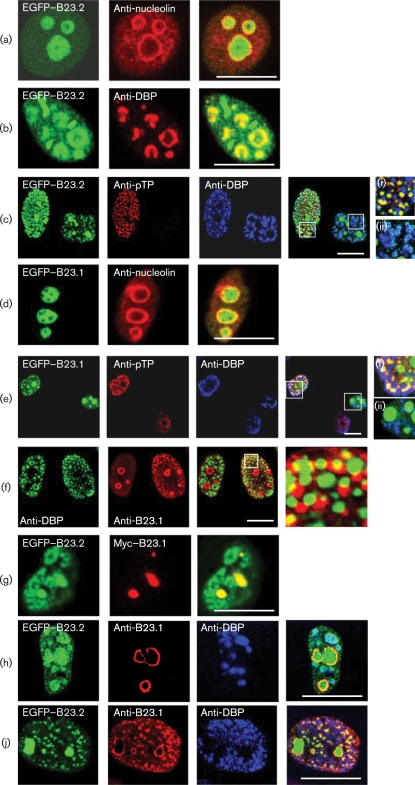
Subcellular location of EGFP–B23.2, EGFP–B23.1, endogenous B23.1, pTP and DBP in uninfected and adenovirus-infected cells. In all images, viral proteins are detected by relevant antiserum. (a) EGFP–B23.2 (green) and nucleolin (a marker for the nucleolus; red) in uninfected cells. (b) EGFP–B23.2 and DBP (red) in adenovirus-infected cells. (c) EGFP–B23.2 (green), pTP (red) and DBP (blue) in adenovirus-infected cells. Note that two cells transfected with EGFP–B23.2 are shown, one expressing both DBP and pTP and the other expressing DBP only. Insets: (i) close-up of the region boxed in the left-hand cell; (ii) close-up of the region boxed in the right-hand cell. (d) EGFP–B23.1 (green) and nucleolin (red) in uninfected cells. (e) Cells transfected with EGFP–B23.1 and infected with Ad2. pTP is shown in red and DBP in blue. Insets: (i) close-up of the region boxed in the left-hand cell; (ii) close-up of the region boxed in the right-hand cell. (f) Endogenous B23.1 (red) and DBP (green) in infected cells. (g) Cell transfected with both EGFP–B23.2 plus Myc–B23.1 (red) and infected with adenovirus. (h) EGFP–B23.2 expression in an infected cell, showing endogenous B23.1 (red) and DBP (blue). (j) EGFP–B23.2 expression in an infected cell, showing extranucleolar endogenous B23.1 (red) and DBP (blue). All images were taken by using a Leica confocal microscope with a ×63 oil immersion lens at the University of Bristol MRC Cell Imaging Facility, Bristol, UK, and are of a single focal plane approximately 0.3 μm deep; bars, 10 μm.

**Fig. 3. f3:**
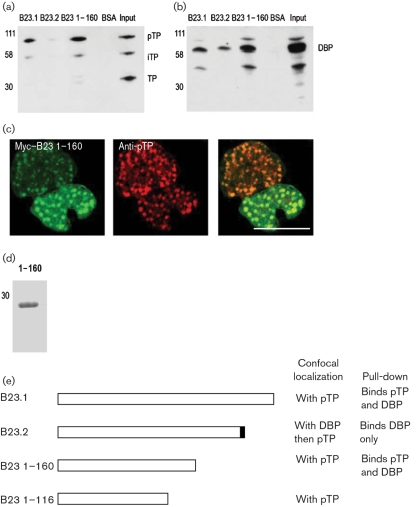
(a, b) Western blot for (a) pTP and (b) DBP following depletion of Ad2-infected cell lysates by B23.1, B23.2, B23 1–160 and BSA. (c) Myc–B23 1–160 and pTP in infected cells. Image was taken by using a Leica confocal microscope with a ×63 oil immersion lens and is of a single focal plane approximately 0.3 μm deep; bar, 10 μm. (d) Purified B23 1–160 was analysed by SDS-PAGE and Coomassie stain for quality. (e) Schematic diagram illustrating the binding and co-localization properties of B23.1, B23.2, B23 1–160 and B23 1–116 in infected cells. Note that the last 2 aa of B23.2 are not equivalent to sequences in B23.1. In the case of B23.1 and B23.2, the co-localization is confirmed by EGFP- and Myc-tagged fusion proteins. For B23 1–160, the localization was shown by Myc-tagged fusion protein only and, for B23 1–116, the co-localization was shown by EGFP-tagged fusion protein only.
